# Dealing with Skin and Blood-Brain Barriers: The Unconventional Challenges of Mesoporous Silica Nanoparticles

**DOI:** 10.3390/pharmaceutics10040250

**Published:** 2018-12-01

**Authors:** Alessandra Nigro, Michele Pellegrino, Marianna Greco, Alessandra Comandè, Diego Sisci, Luigi Pasqua, Antonella Leggio, Catia Morelli

**Affiliations:** 1 Department of Pharmacy and Health and Nutritional Sciences, University of Calabria, 87036 Rende, Italy; nigroale16@gmail.com (A.N.); michele.pellegrino@unical.it (M.P.); mariannagreco.89@gmail.com (M.G.); alessandracomande@outlook.it (A.C.); diego.sisci@unical.it (D.S.); 2 Department of Environmental and Chemical Engineering, University of Calabria, 87036 Rende, Italy; luigi.pasqua@unical.it

**Keywords:** mesoporous silica nanoparticles (MSNs), drug delivery system (DDS), topical drug delivery (TDD), blood-brain barrier (BBB)

## Abstract

Advances in nanotechnology for drug delivery are fostering significant progress in medicine and diagnostics. The multidisciplinary nature of the nanotechnology field encouraged the development of innovative strategies and materials to treat a wide range of diseases in a highly specific way, which allows reducing the drug dosage and, consequently, improving the patient’s compliance. Due to their good biocompatibility, easy synthesis, and high versatility, inorganic frameworks represent a valid tool to achieve this aim. In this context, Mesoporous Silica Nanoparticles (MSNs) are emerging in the biomedical field. For their ordered porosity and high functionalizable surface, achievable with an inexpensive synthesis process and being non-hazardous to biological tissues, MSNs offer ideal solutions to host, protect, and transport drugs to specific target sites. Extensive literature exists on the use of MSNs as targeted vehicles for systemic (chemo) therapy and for imaging/diagnostic purposes. However, the aim of this review is to give an overview of the last updates on the potential applications of the MSNs for Topical Drug Delivery (TDD) and as drug delivery systems into the brain, discussing their performances and advantages in dealing with these intriguing biological barriers.

## 1. Introduction

Nanotechnology emerged as one of the most promising technologies in the 21st century, representing a multi-disciplinary approach applying engineering and biology principles at the molecular level. Nanotechnologies refer to the design of small but very smart and flexible structures, whose application covers different fields, ranging from electronics to information technology, energy, environmental science, transportation, biomedical research, food industry, and home and personal care. In particular, nanobiotechnology holds great potential in life science and it is increasingly attracting the attention of scientists all over the world. For instance, in the pharmaceutical field, smart nanostructures revolutionized how drugs are formulated and delivered (e.g., hosting old drugs in new packaging which are able to selectively recognize the target tissue), paving the way to nanomedicine, that marks an unparalleled opportunity to advance the treatment of various diseases, including cancer [1]. In this context, several nanostructured organic and inorganic functional materials are emerging as excellent candidates for delivery of a wide range of drugs [2]. Among them, Mesoporous Silica Nanoparticles (MSNs) have become a novel generation of inorganic framework for biomedical applications [3,4,5] due to their tunable size (2–50 nm), high surface area (700–1000 m^2^/g), well-ordered internal mesopores (typically 2–6 nm), large pore volume (0.6–1 cm^3^/g), high drug loading, good biocompatibility, and low production costs [6]. In addition, it is possible to chemically modify MSNs’ surface properties through covalent grafting of various organic functional groups to the free silanol groups. These advantageous features make MSNs ideal platforms to design multifunctional nanosystems, boasting eligible qualities for high drug loading and gradual release [7]. Vallet-Regí M et al. were the first to develop ordered mesoporous materials carrying the drug ibuprofen as drug delivery systems (DDSs) [8]. Since then, MSNs have been tested for the delivery of a variety of drugs including nonsteroidal anti-inflammatories (e.g., aspirin), antibiotics (e.g., vancomycin), and chemotherapeutics (e.g., doxorubicin and methotrexate) [9,10,11,12].

The big advantage of these nanomaterials is that they can be tailored to continuous or triggered release of a wide range of molecules, depending on the application. For instance, Sanchez-Salcedo S. et al. recently proposed new multifunctional polyethylenimine (PEI) coated core-shell Fe_3_O_4_@SiO_2_ MSNs, with a zwitterionic 2-methacryloyloxyethyl phosphorylcholine (MPC) surface, aimed at reducing unwanted protein (corona proteins) adsorption on the MSNs surface once entered the bloodstream, thus minimizing opsonization and prolonging MSNs blood circulation time. The device also carries an anti-TWIST siRNA, which, knocking down TWIST expression, sensitizes ovarian cancer cells to the action of the other cargo molecule, daunorubicin, whose release is triggered by externally applied oscillating magnetic fields (OMF) [13].

Notably, drug release control by means of opportune gatekeepers (e.g., pH, magnetic fields, light, temperature, reducing agents) can be so efficiently designed to achieve “zero premature release” [14]. Moreover, surface functionalization with targeting ligands [15] is the most exciting way to deliver the drug to target cell, performing an extremely selective therapy [16]. In this context, Lopez V. and co-workers developed Janus MSNs asymmetrically decorated with two targeting functions, folic acid (FA) to selectively recognize the folate receptors (FR) on the cell membranes and triphenylphosphine (TPP) to specifically target mitochondrial membranes, with the aim of improving the therapeutic efficacy [17]. Particularly fascinating is also the totally innovative approach of using human Decidua Mesenchymal Stem Cells (DMSCs) as carriers for doxorubicin (DOXO) loaded MSNs to direct the delivery system exclusively to the tumor site, exploiting DMSCs migratory attitude towards tumors [18].

In addition, unlike organic nanocarriers (e.g., nanocapsules, liposomes, polymeric micelles and NPs, protein NPs), characterized by physico-chemical instability, low encapsulation efficiency, premature drug leakage, and lack of tunable triggers for drug release [19], and unlike certain inorganic nanomaterials that are not biodegradable and/or toxic, MSNs’ own important properties make them ideal platforms for biomedical applications. In particular, they exhibit excellent biochemical and physico-chemical stability, excellent drug delivery and endocytotic behaviors, and, most importantly, high biocompatibility and degradability [3]. In fact, MSNs are characterized by a hydrolytically unstable framework, that, once in the bloodstream, is rapidly converted by hydrolysis into water-soluble silicic acid (Si(OH)_4_), which, in turn, is safely excreted through the urines [20]. Moreover, it is well established that, due to wide range of synthesis procedures and the resulting physical characteristics, general assumptions about MSNs potential toxicity and organ accumulation cannot be drawn. In fact, MSNs degradability and toxicity is affected by different parameters such as size (MSNs of 30 to 100 nm diameters are able to induce inflammatory response in animal models [21]), morphology, porosity, surface charge (e.g., anionic surfaces are generally less toxic than cationic NPs, which can cause hemolysis [22]), and functionalization. Therefore, in order to develop safe silica nanoparticles carriers, a fine-tuning of these structural characteristics is mandatory.

For all these peculiarities, MSNs have found potential application in targeted therapy, both in cancer treatment and in diagnosis. Indeed, multifunctional MSNs are currently being applied in the bio-sensing field and as ideal cell tracing in the detection of analytes within individual cells both in vitro and in vivo [23]. Since nanoparticles (NPs) do not suffer from fluorescent self-quenching, and other diffusion-related problems, they can be functionalized with large quantities of cell-recognition or other site-directing compounds. So, fluorescence-traceable MSNs are useful tools for cell tracking via fluorescence microscopy [24]. Likewise, magnetic resonance imaging (MRI)-traceable MSNs are employed in both clinical- and research-based fields, due to their deep tissue imaging capabilities [25].

To date, MSNs have been explored for simultaneous imaging and therapy, making them a biomedical platform for theranostic applications. MSNs have emerged as suitable for long-term quantitative imaging at low doses, since they are safely cleared from the body after imaging is complete [26]. It is also noteworthy that MSNs exhibit low hemolytic activity, confirming their suitability for systemic delivery through the bloodstream [27]. Not so long ago, dye-doped silica NPs, called Cornell dots (C dots), have been approved from the FDA for the first Investigational New Drug (IND) application for targeted molecular imaging in the oncology field [28,29].

However, beyond these “conventional” uses, MSNs have also attracted great interest in the dermatological and neurological fields. Therefore, the purpose of this review is to offer a brief, but exhaustive, overview on MSNs’ applications in the topical and neurological treatments, focusing the attention on MSNs’ interactions with biological barriers, such as skin and the blood-brain barrier (BBB).

## 2. Drug Delivery Systems (DDSs)

Drug delivery is the approach of administering therapeutic agents in formulations, technologies, and systems in order to safely achieve the desired therapeutic effect by decreasing fluctuations in serum drug concentrations, which results in reduced toxicity, sustained efficacy, and less frequent dosing.

The main advantage of DDSs is that the drug is isolated from the bloodstream along its way, it does not interact with non-target cells, and it is released only when the desired site has been reached. This implies that the therapeutic efficacy is guaranteed by even lower doses of the encapsulated drug, thus reducing the side effects, which still represent the main limitation of the conventional formulations [2].

To fulfill this need, the delivery of drugs is often committed to smart NPs that are able to recognize the target organs, tissues, and cells, overcoming problems such as limited solubility, molecular aggregation, poor biodistribution, and lack of selectivity [30].

Different features make an efficient DDS: biocompatibility, high loading/encapsulation of desired drug molecules, absence of premature release, cell type or tissue specificity, and stimuli-responsivity (e.g., pH- or temperature-sensitive). The possibility of functionalizing the surface by conjugating target molecules (e.g., ligands) allows one to direct the loaded drug exclusively to the site of interest, exploiting the molecular recognition of target receptors [31,32].

MSNs possess all the characteristics of an efficient DDS [2], so they are emerging as good candidates to become reliable vehicles in a variety of applications [5,33,34].

## 3. MSNs in Topical Drug Delivery

The skin consists of four layers: the stratum corneum (SC), which is the outermost layer of the skin, the epidermis, the dermis, and the subcutaneous tissues (hypodermis). A number of appendages (i.e., hair follicles, sweat glands, and nails) are also associated to the skin. The SC represents the main barrier to drug permeation, consisting of a cornified cell envelope, composed of high density and low hydrated layers of flat and elongated corneocytes and a densely packed protein/lipid polymer structure right below the cornified cell envelope. The other layers and the appendages, although more permeable, offer several target sites for drug delivery [35].

Topical Drug Delivery (TDD) involves the use of drugs to be applied directly on the skin in a formulation that can be absorbed. To reach systemic circulation, the drug has to permeate the above described consecutive skin layers, i.e., once released from the TDD the drug penetrates into the SC, then passing to the more aqueous epidermis and, finally, absorbed by the capillaries in the dermis.

The use of TDD could potentially overcome the conventional routes of administration, oral and parenteral, still guaranteeing a comparable efficacy, but circumventing issues such as systemic side effect and needle phobia. Skin patches are among the first examples of topical drug delivery systems [36]. Therapeutic concentrations of topically delivered agents accumulate within the site of application, maintaining low serum levels, thus resulting in less organ toxicity [37].

In order to cross the multilayer barrier of the skin, it would be desirable to administer the chemical agents through carriers able to locally deliver the drug and to store it into the skin appendages. Among the different carriers proposed, silica nanoparticles gathered a wide consensus [38,39,40].

**Figure 1:**
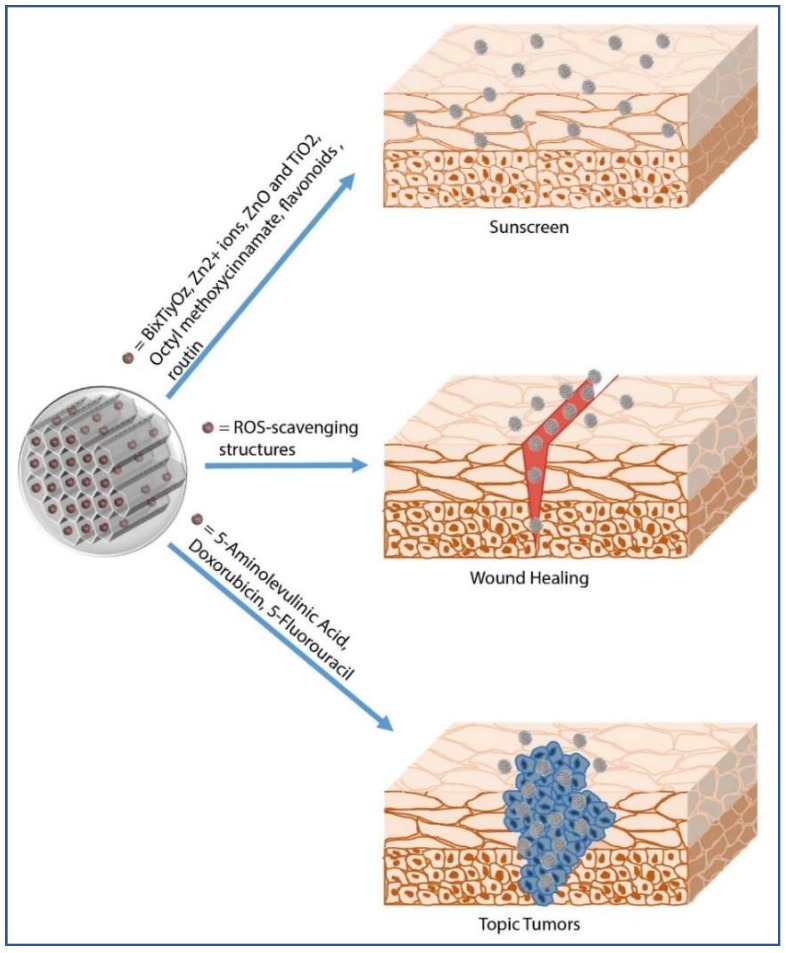
MSNs as topical drug delivery systems.

Chemicals can be absorbed by the skin only after having overcome its constituent layers, either via the intercellular route, with partitioning into the lipid matrix, or via the intracellular route, or through sweat glands or hair follicles [41]. In particular, some evidences suggest that the hair follicle may constitute a significant reservoir for topically applied molecules [42] and NPs [43,44]. In fact, their functional location could mediate the diffusion across the capillary walls of the stored molecules to the surrounding spaces. Therefore, these structures may represent a potential target for nanomaterial-based shuttles, as reported by Xuan et al., who showed accumulation of fluorescent MSNs in the hair follicles of porcine ear skin [44]. However, toxicological issues on transdermal delivery of nanomaterials are still under debate [44,45,46]. It is well established that size, shape, surface charge, *z* potential, and tendency to aggregate are crucial parameters affecting the interactions of NPs with human skin surface. Generally, only NPs below 1 nm are able to permeate the intact skin, while silica NPs with an average size lower than 25 nm can penetrate but not permeate the skin [44] and those with higher diameters (e.g., 55 ± 6 nm) are not able to cross the normal or perturbed mouse skin after one or five days of topical application [44]. Examples of therapeutic molecules topically delivered by means of silica NPs include corticosteroids, antifungals, antivirals, antibiotics, antiseptics, local anesthetics, antineoplastics, as well as antioxidant molecules included in cosmetic products (e.g., moisturizers, make-up, sunscreen etc.). Table 1 and Figure 1 summarize multiple uses of silica NPs in topical drug delivery.

## 4. MSNs in Cosmetics

Many substances currently proposed for topical drug delivery are being questioned for their adverse health and environmental effects, thus promoting the search for safer alternatives for human health. An example are the inorganic ingredients of sunscreens, used to preserve skin from UV radiation. In this context, silica NPs offer solutions which are highly compatible with human health and the environment [33].

Indeed, Sotiriou et al. increased the safety of sunscreen constituents (ZnO and TiO_2_) by coating the surface of these hazardous nanoparticles with silica layers [58]. Their toxicological data showed that the hermetic encapsulation of the ZnO nanorods in a thin silica shell minimized the bio-interactions of the core ZnO, in particular reducing the strong DNA damage otherwise observed with the pure uncoated ZnO nanorods [58]. The silica coating is not only useful to protect the skin against the toxicity of these UV filters, but also to improve their stability within the sunscreen formulation.

Knežević et al. [33] emphasized the high potential of functionalized silica-based nanomaterials (healthier and environmentally friendly) for application in skin protection from UV irradiation. Tolbert et al. have designed hybrid organic/inorganic silica particles, encapsulating organic sunscreens in silica NPs, in order to reduce their phototoxicity and degradation [59]. Similarly, the octyl methoxycinnamate, one of the most common UVB filters included in sunscreen formulations, when encapsulated in silica scaffold, improve its photostability [47], corroborating the usefulness of silica nanosystems in the cosmetic field.

Antioxidants like flavonoids are frequently used in sunscreen formulations to complement UV filter photoprotection [51]. MSNs have been proposed as an innovative topical carrier, able to preserve the physico-chemical and biological properties of labile active ingredients of dermocosmetic interest until their release on the skin. Ugazio et al. [48] demonstrated that the antioxidant efficacy of quercetin was maintained upon immobilization in the thermosensitive silica NPs. Furthermore, the association of quercetin to aminopropyl-functionalized silica NPs leads to 2-fold increase in skin penetration, compared to free quercetin, after 24 h of application [40,51,60]. The use of nanodosage forms of quercetin is promoted in various skin disorders, such as atopic dermatitis or psoriasis, as well as in sunscreen formulations. In fact, these innovative nanosized quercetin complexes show improved skin permeation compared to the free quercetin, whose topical penetration is hindered by its poor water solubility and low stability. In addition, the antiproliferative activity of quercetin-loaded (Q-loaded) MSNs have been evaluated in JR8 melanoma cells [51], confirming that the Q-nanosystems enhance the flavonoid cytotoxicity, compared to the free form, probably for an increase in the antioxidant release or in the cellular uptake of the nanosized system.

Other agents, such as routin, used in cosmetics for UV protection and for their antioxidant properties, have been incorporated in the MSNs cavities in order to improve their photostability and optimize their biological function [47,52].

These studies pave the way for an innovative employment of mesoporous silica materials in the skin care field and in topical products.

Recently, silica NPs have been proposed as a promising scaffold for the wound healing process due to the interactions occurring between the material surface and the tissue matrix, a nanobridging effect that promotes a rapid sealing of the wound [49]. Moreover, when the excellent tissue adhesive ability of MSNs is coupled to the delivery of active ingredients to damaged tissues the result is even better. In fact, the synergistic effect of nanobridging and ROS-scavenging structures, as in ceria nanocrystals decorated MSNs (MSN-Ceria), accelerates the wound healing process by reducing the oxidative stress of the microenvironment, thus improving the regeneration process of the injured skin and limiting scar formation [49]. These data were also confirmed in vivo, using a cutaneous wound rat model. MSN-Ceria nanostructures display the same adhesive strength as MSNs alone, but promote structure restoration of wounded skin due the ROS-scavenging activity. Treatment with this combination mended the wound area almost completely within 8 days, achieving the smooth appearance of the damaged skin area. Therefore, Ceria-MSN led to a significantly enhanced therapeutic effect and improved the quality of the healed skin [49].

The versatility of nanomodels like MSNs is also beneficial in the oral hygiene field. In fact, fluorescent MSNs can be uptaken, degraded, and/or excreted by the reconstructed human gingival epithelia (RHGE) [61], accumulating at the superficial corneum layer in a time-dependent manner and forming a “nanocoating-like barrier”. So, nanoparticle-based antimicrobial and anti-inflammatory agents could be employed for topical application in oral healthcare [61].

## 5. MSNs in the Topical Treatment of Cancer

Extensive literature is available on the use of targeted silica and silica-based NPs in cancer treatment, in order to overcome the well-known limitations of conventional chemotherapy due to high systemic exposure to anti-neoplastic agents that frequently results in dose-limiting toxicity [3,34].

The topical administration of anticancer drugs through nanoparticles-based delivery systems is an interesting alternative to the systemic skin cancer treatment for improving drug targeting and therapeutic benefits. Nanocarriers are not only useful to protect antitumor drugs against degradation, but also to enhance drug penetration into the deep layers of the epidermis. Furthermore, TDDSs significantly reduce antitumor drug side effects, as they do not directly enter into the bloodstream. Indeed, in vitro preliminary findings suggest that MSNs could represent a promising strategy and the most innovative technology in the topical application of anticancer therapies.

Anirudhan et al. [56] engineered a temperature- and ultrasound (US)-sensitive hybrid MSNs system (HMSN) for the delivery of 5-fluorouracil (5-FU), an anticancer drug. The NPs were developed by grafting the MSNs’ pores with a temperature and US sensitive copolymer of tetrahydropyranyl methacrylate (THPMA) and amino ethyl methacrylate (AEMA). Exposing the 5-FU loaded HMSNs to US frequency, the acetal bonds of THPMA moiety are cleaved with the consequent release of the encapsulated drug molecules. The US frequency also acts as a permeation enhancer, allowing controlled drug permeation across the skin barrier.

Methotrexate (MTX) is a chemotherapeutic agent employed in the treatment of different types of cancers and inflammatory processes, but also in bowel and Crohn’s diseases, psoriasis, and other skin disorders. Like any other chemotherapeutic agent, MTX activity associates to a series of unwanted effects, which can be overcome by the use of nanotechnology. Recently, Sapino et al. [38] have developed a series of formulations containing MTX-loaded (by absorption) MSNs for the topical administration of methotrexate (MTX). The MTX/MSN system increases the epidermal accumulation of the active molecule and allows delivering the drug to the deeper layers of the epidermis.

A promising drug delivery system that combines chemotherapy and photothermal therapy (PTT) has been proposed for the treatment of superficial tumors. The system (PVP@DOX/MSN@ICG) is composed of microneedle (MN) patches loaded with the chemotherapeutic drug Doxorubicin (DOX) and covalently conjugated to the photothermal indocyanine green (ICG), to enhance ICG stability and to maintain its photothermal efficiency. DOX and ICG are released in the tumor site at the same time by dissolution of the microneedles in the extracellular fluid [53].

The selectivity of the nanoparticles against the tumour cells is improved by decorating MSNs’ external surface with targeting molecules that specifically bind to receptors on the cell surface of interest to promote nanocarrier interaction and internalization. To date, only a few, but significant, examples of targeted MSNs have been developed for topical application in cancer treatment. Martìnez-Carmona et al. [55] proposed a novel nanocarrier based on mesoporous silica particles covered with a photosensitive protein shell, which is cleavable by light irradiation. Transferrin (Tf) molecules anchored through a UV-sensitive cross-linker on the surface of Doxorubicin (DOXO)-loaded MSNs, act as targeting agents and cleavable gatekeepers at the same time. The in vitro cytotoxicity of the MSN-Tf-DOXO system, evaluated on various tumor cell lines overexpressing transferrin receptors, showed excellent performance of the system. The developed carrier could be applied to the treatment of tumors easily accessible for direct light irradiation, such as skin cancer.

Photodynamic therapy (PDT) is a noninvasive cancer therapeutic technique that uses a photosensitizer and a particular type of light. The photosensitizer, upon exposure to a specific wavelength of light, converts surrounding oxygen into singlet oxygen, a form of reactive oxygen species that kills tumor cells. Ma et al. [54] realized a multifunctional MSN-based delivery system of 5-Aminolevulinic Acid (5-ALA), a precursor of the photosensitizer protoporphyrin IX (PphIX), for skin cancer therapy through the PDT process. The developed carrier consists of 5-ALA-loaded MSNPs externally functionalized with the targeting ligand folic acid (FA) that allows receptor-mediated endocytosis of the system into cancer cells and the polymer polyethylene glycol (PEG) that enhances its biocompatibility. PphIX formed from 5-ALA@HMSNP-PEG+FA upon light irradiation showed high photocytotoxicity to the cancer cells in vitro.

## 6. Drug Delivery as a Potential Approach to Cross the Blood-Brain Barrier (BBB)

The proper delivery of drugs to the central nervous system (CNS) is often hampered by the cunning nature of the blood-brain barrier (BBB) that serves as a critical barrier between the CNS and the peripheral circulation, maintaining the brain’s homeostasis and preserving its internal milieu [62]. Thus, although protecting the brain from noxious agents, BBB is also an obstacle to the delivery of beneficial drugs to treat CNS diseases [63,64]. Compared to other endothelial cells, the brain capillary endothelial cells (BCEC) show some features (e.g., the lack of fenestrations, the circumferential tight junctions complexes, minimal trancytosis, diminished pinocytosis and para- and transcellular barriers consisting of cell membranes, various enzymatic filters, and efflux transporters) which contribute to protecting the brain from toxic agents [65]. Around 100% of molecules over 500 Da and 98% of small molecules are not able to penetrate the brain after systemic administration and alterations in the BBB permeability occurring in pathological conditions such as Alzheimer’s (AD) and Parkinson’s (PD) diseases and stroke may limit the delivery of drugs into the CNS even more [66]. Therefore, new routes of drug administration able to transiently induce BBB disruption have been suggested, including intracerebral pathways, blood-to-brain delivery, and intranasal delivery coupled to biological, chemical, or physical stimuli such as zonula occludens toxin, mannitol, magnetic heating, and ultrasound. However, these approaches showed a series of disadvantages, such as being dangerous, expensive, and unsuitable for the treatment of most brain diseases and for the delivery of most drugs [67]. Therefore, the ideal method to transport drugs across the BBB should be controlled and not damage the barrier. Among the different approaches proposed, nanobiotechnology-based delivery methods provide the best prospects to achieve this ideal. The strategy of vector-mediated blood-to-brain delivery, aimed at increasing BBB permeability to the drug-carrier conjugate, can minimize potential injuries to the barrier. Being submicrometer objects that behave as a whole unit in terms of transport and chemical properties, nanomaterials are promising vehicles for direct drug transport across the intact BBB. This is due to their potential to enter the BCEC by means of normal endocytosis and transcytosis, thanks to their small size, as well as their high versatility, since they can be functionalized with multiple copies of the drug molecule and the targeting function of interest [63,64].

Different types of nanocarriers have been extensively studied for drug delivery to the brain: liposomes, polymeric (synthetic and natural) and inorganic NPs (e.g., silica based, carbon nanotubes and gold NPs). Compared to synthetic polymeric NPs, the inorganic NPs offer a series of advantages, i.e., the control over shape and size as well as the ease of preparation and functionalization. Moreover, they can be easily detected by microscopy and analytic techniques (e.g., MRI, TEM, and ICP-MS). Although natural NPs (chitosan, alginate, poly(lysine), gelatin, and albumin) have the advantage of triggering biological signals by interacting with specific receptors/transporters expressed by BCEC, compared to inorganic NPs, they show lower versatility, higher batch-to-batch variability, and poor tracking capacity by imaging techniques. [66]. However, common issues in the use of inorganic NPs reside in their potential toxicity, organ accumulation, and degradation. In this context, although MSNs’ application as potential drug delivery systems have been largely exploited in the biomedical field, mainly for systemic administration, there is still no actual evidence on the toxic effects at the BBB level [67].

**Figure 2:**
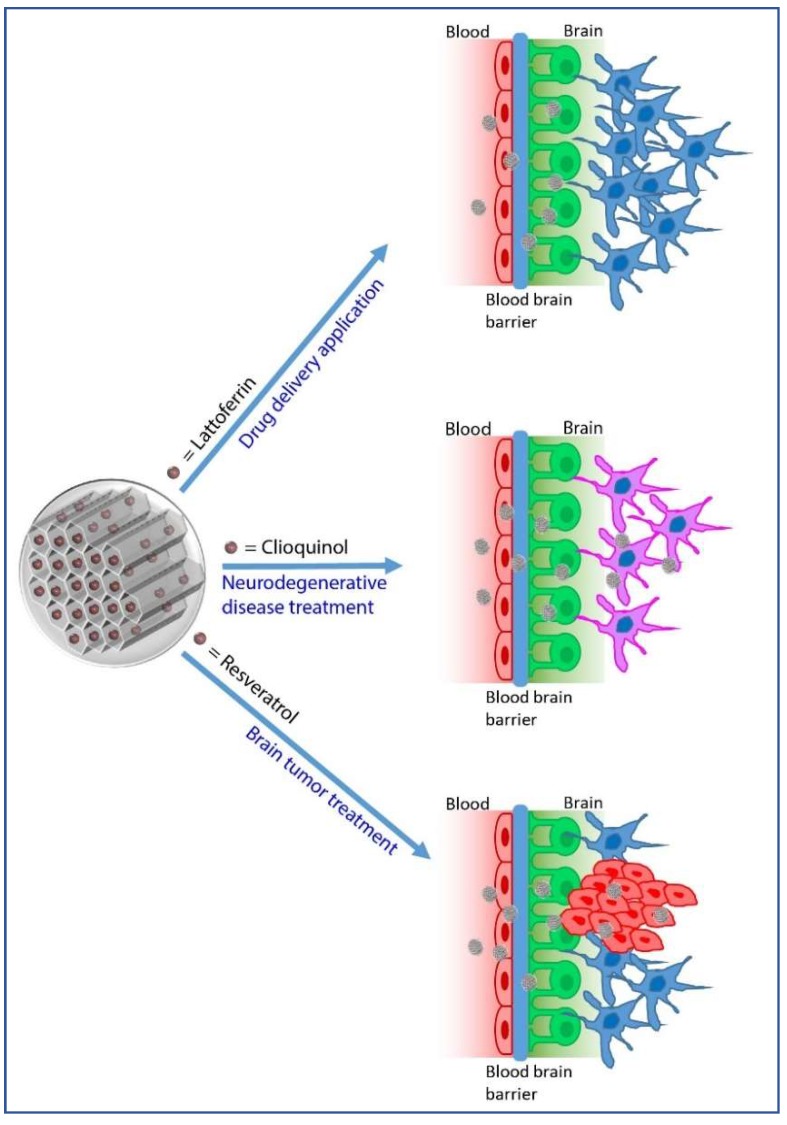
Main applications of MSNs as drug delivery systems for brain related diseases.

The main applications of silica NPs as drug delivery systems for brain related diseases are reported in Table 2 and Figure 2.

## 7. MSNs as Drug Delivery Systems Targeting the BBB

In last two decades, a series of drug delivery systems targeting the BBB have been tested with various degrees of success. Recently, several studies demonstrated that NPs’ transcytosis across the BBB occurs through receptor-mediated transcytosis (RMT), a selective and non-invasive delivery mechanism. For example, in the vascular endothelial cells (VECs), RMT seems to occur by means of different receptors, including the transferrin receptor (TfR) and the low-density lipoprotein receptor (LRP). In addition, ligand-bearing liposomal or polymeric nanocarriers can also promote the delivery of drugs through the BBB [76,77,78,79,80]. However, these approaches did not translate to clinical practice for their poor delivery efficacy; therefore, developing alternative strategies is still mandatory.

To overcome the problem, Yang S. and colleagues designed a new drug carrier, consisting of silica NPs that exploit endogenous transcytosis pathway for effectively delivering therapeutics without disrupting the normal function of BBB [69].

In this work, the authors showed that fluorescence dye and polyethylene glycol (PEG) bearing MSNs grafted with the glycoprotein Lactoferrin (Lf) are able to cross the BBB (mimicked by in vitro co-cultures platforms) and that the efficiency of transcytosis increases as the particle size decreases [69]. Moreover, being highly expressed in human VECs of the BBB, Lactoferrin (Lf) ligands are more efficient and specific than those targeting the transferrin (Tf) receptor [68,81,82,83,84,85,86]. Besides, considering also their good biocompatibility and relatively low cost, Lf ligands represent promising candidates for BBB targeting [69].

## 8. MSNs-Based Therapy in Alzheimer’s Disease

Alzheimer’s disease is the most common form of dementia and its incidence is greatly increased as a result of the general aging of the population worldwide. The etiology of this pathology is currently based on two different hypotheses: (1) the aggregation and accumulation of amyloid-β (Aβ) peptides, typically leading to fibrils in the brains of patients and (2) the “tau hypothesis”, referring to the abnormal aggregation into masses of tau microtubule-associated proteins in conjunction with amyloid plaques [87].

Among various therapeutic NPs based prototypes, designed with the aim of inhibiting the Aβ aggregation occurring in the Alzheimer’s disease, MSNs offer the advantage, if opportunely functionalized, of selectively crossing the BBB, delivering relatively high payloads of cargo molecules into the brain [6].

Qu and co-workers observed that metal ions (e.g., Cu^2+^ ions) limited Aβ solubility by chelating histidine residues, promoting Aβ aggregation and inducing the formation of cytotoxic ROS (H_2_O_2_). Therefore, they engineered H_2_O_2_-responsive MSNs (MSN–Cu@IgG) coated with immunoglobulin G (IgG) in order to release the metal chelator clioquinol (CQ) upon redox actuation, thus inhibiting Aβ fibril formation [70]. In addition, the treatment with the nanovector increases cell viability, emphasizing its potential use in Alzheimer’s disease.

Yang et al., exploiting the metal chelating characteristics of CQ and the ability of gold NPs to inhibit Aβ aggregation, developed H_2_O_2_-responsive mesoporous silica-based vectors capped with gold nanoparticles and bearing CQ [71]. Ganji and collaborators designed MSNs for the delivery of rivastigmine hydrogen tartrate, a drug widely used in the treatment of Alzheimer’s disease, into human SY5Y neuroblastoma cells [72].

## 9. MSNs-Based Therapy to Cure Brain Tumors

Brain tumors affect approximately 5 to 10 people per 100,000 and are considered as one of the 10 main causes of death by cancer [88]. The standard treatment for glioma, a malignant tumor of nervous system tissue, typically involves surgical resection followed by a combination of radiation and chemotherapy, but this approach has not substantially improved the overall survival (the median survival period remains ~16 months, while the five-year survival rate is ~10%) [89].

In particular, the blood-brain barrier (BBB) and blood–tumor barrier (BTB) hamper the tumor penetration and uptake, which makes the treatment with most therapeutic agents for glioblastoma multiforme (GBM) particularly inefficient [90,91]. Therefore, cancer-targeted drug delivery systems with permeability of the blood–brain barrier (BBB) have become of great interest for the rational design of high-efficiency anticancer agents. Recently, You Y. and colleagues reported a strategy for the rational design of a tailored nanomedicine with enhanced BBB permeability to treat human brain glioma [75]. The authors designed and tested a new tailored MSNs nanosystem as a carrier for anticancer agents, using a novel organic selenium compound BSeC as a model molecule and the tripeptide RGD (arginine–glycine–aspartate) as targeting molecule, recognizing a subset of the integrins on tumor membranes [74]. The results showed that this nanosystem (BSeC@MSNs-RGD) exhibited high stability in human blood serum and could effectively transport across the BBB, thus enhancing the permeability of loaded drugs against BBB. In addition, this tailored MSNs also enhanced the cellular uptake of BSeC, thus significantly increasing its anticancer efficacy by a factor of hundreds of times by the induction of cell apoptosis. BSeC@MSNs-RGD selectively entered cancer cells, which express high levels of integrin molecules, compared to normal cells. The internalized BSeC@MSNs-RGD triggered mitochondrial dysfunction and intracellular ROS overproduction, which subsequently activated the p53 and MAPKs pathways to promote cell apoptosis. The nanosystem also showed excellent penetrating ability and inhibitory effects on human glioblastoma cell line U87 spheroids, supporting its in vivo anticancer potential. Furthermore, the tailored MSNs nanosystem significantly prolonged the blood circulation time of loaded drugs in vivo and was easily cleared by renal excretion, thus effectively reducing toxicity.

Mo J. and co-workers tailored the size of MSNs loaded with the chemotherapeutic doxorubicin (DOX) and modified by the cancer targeting polymer PEI-cRGD (poly(ether imide)-cricoids Arg-Gly-Asp-Phe-Lys). The functionalized nanosystem (DOX@MSNs) selectively recognized overexpressing ανβ3 integrin U87 glioblastoma cells. Exposing the cells to MSNs ranging from 40 to 120 nm in diameter, the authors observed that the cellular uptake and the consequent inhibition of glioma cells were proportionally higher with the decrease of the particle size. In particular, the 40 nm DOX @ MSNs not only rapidly enter tumor cells, but also show a greater selectivity and antitumor activity compared to free Dox, inducing apoptosis in glioma cells through ROS overproduction. Moreover, the 40 nm DOX @ MSNs showed increased ability in overcoming the BBB, also interfering with the vasculogenic mimicry (VM) capacity of glioma cells by altering MMP-2, E-cadherin, and FAK proteins expression. This feature allows achieving a satisfactory antitumor efficacy, while avoiding unwanted toxicity to normal brain tissue [75].

## 10. Neurodegenerative Disease and MSNs Therapy

It has been well established that the accumulation of reactive oxygen and nitrogen species (RONS) are responsible for neuronal injury and inflammation, leading to various neurodegenerative disorders. A large number of studies evidenced that an antioxidant-based therapy can be effective to ameliorate the deleterious effects of RONS [92]. In this context, the antioxidant compound Resveratrol (RSV) is currently under investigation for its antioxidant properties, as it is able to remove the excess of RONS generated in the brain. However, since very low amount of RSV reach the CNS after systemic administration, Shen Y. et al. have recently proposed a new delivery system consisting of MSNs coated with polylactic acid (PLA) and conjugated with a ligand peptide recognizing the low-density lipoprotein receptor (LDLR). As conceived, the system strongly enhances RSV transcytosis across the BBB. The PLA coating prevented the RSV premature release, which occurred only in presence of high ROS levels that are able to accelerate PLA degradation. The RSV delivery across BBB was evaluated using a co-culture of rat brain microvascular endothelial cells (RBECs) and microglia cells, which mimic an in vitro BBB model. The conjugation of LDLR ligand peptide markedly enhanced the migration of MSNPs across the RBECs monolayer and RSV could be released and effectively reduce the phorbol-myristate-acetate or lipopolysaccharide mediated activation of the microglia cells [73].

Several phytochemicals are known to protect neurons from injuries occurring during neurodegenerative diseases [93,94,95,96,97,98,99]. Currently, drug delivery into the brain after systemic administration is limited by the poor permeability of the BBB. Thus, a promising alternative administration route to bypass the BBB is represented by the nose-to-brain delivery. Lungare S. and co-workers proposed MSNs bearing the poorly water-soluble phytochemicals chrysin and curcumin (MSNP) as potential drug delivery systems that exploit olfactory targeting for the treatment of neurodegenerative diseases. The authors show that small MSNP particles (below 500 nm) could be endocytosed by olfactory cells and that the slightly acidic pH of the nasal cavity can trigger the pH-dependent release of the phytochemicals. Overall, this drug delivery system targeting the BBB would represent an interesting alternative to the systemic administration, since a lower drug cargo is needed to achieve similar therapeutic effects [62].

## 11. Conclusions

Nanotechnologies have gained attention in all areas of science and, among the nanomaterials, MSNs boast considerable potential due to their unique properties. This review is an update on the advantages and the potential applications of MSNs as TDD and as drug vehicles opportunely tailored to overcome the BBB. Although a series of very interesting preclinical studies have been conducted, demonstrating MSNs’ ability in crossing both skin and the BBB, none of the proposed formulations have yet been tested in clinical trials. The main obstacle to reaching the clinic lies in MSNs’ main features (e.g., surface functionalization, charge, size, and shape) and on the difficulties in the reproducibility of a small-scale synthesis during scale-up processes under Good Manufacturing Practices (GMP) conditions. Moreover, although MSNs showed remarkably high biocompatibility in several in vivo studies, safety issues still have to be properly addressed, conducting crucial evaluations of MSNs pharmacokinetics, clearance, half-life, pharmacodynamics, immunogenicity, and organ accumulation. Therefore, MSNs’ future challenge is to overcome all these limitations in order to eventually start offering forefront and cost-effective solutions that satisfy many different clinical and cosmetic needs.

## Figures and Tables

**Table 1:** Multifunctional Silica Nanoparticles (MSNs) as a versatile platform for Topical Drug Delivery.

Application	Structure	Features	References
**Cosmetics**	Bismuth titanates (Bi_x_Ti_y_O_z_) NPs embedded into MSNs.	Inorganic sunscreen UV filters.	[39]
	MSNs and periodic mesoporous organosilica NPs functionalized with a chelating ligand and Zn^2+^ ions and containing bridging benzene and ethane moieties.	Photostable and safe sunscreen UV filters.	[33]
	Octyl methoxy cinnamates molecules encapsulated into Hollow Silica NPs.	Sunscreen UV filters.	[47]
	MSNs functionalized with *N*-isopropylacrylamide a thermoresponsive copolymer, 3-(methacryloxypropyl)trimethoxysilane inside the mesopores and loaded with quercetin.	Topical carriers for quercetin, antioxidant and labile active ingredients of dermocosmetic interest.	[48]
**Biomedical**	Ceria nanocrystals immobilized onto the surface of MSNs	Tissue adhesive capability and in vivo ROS-scavenging activity	[49]
	MSNs and fluorescent MSNs.	Potential MSN-based anti-infective and anti-inflammatory agents in topical applications for effective oral healthcare.	[44]
	Spherical Colloidal MSNs with ordered mesopores.	Strong adhesives for hydrogels and biological tissues.	[50]
	MSNs and Methotrexate complex.	Dermal delivery of Methotrexate for the treatment of skin diseases.	[38]
	MSNs loaded with quercetin.	Potential topical carrier to load flavonoids derivatives.	[51]
	MSNs covalently coated with antioxidant molecules, caffeic acid or rutin.	New carrier with antioxidant properties.	[52]
**Cancer**	Transcutaneous delivery plat form consisting of Doxorubicin hydrochloride and indocyanine green conjugated with silica NPs loaded on microneedle patches.	Treating superficial tumors using a combination of chemotherapy and photothermal therapy.	[53]
	Multifunctional hollow MSNs containing PEG and folic acid targeting ligand and loaded with 5-Aminolevulinic Acid.	Potential in photodynamic skin cancer therapy.	[54]
	MSNs loaded with Doxorubicin and decorated with a biocompatible protein shell cleavable by light irradiation.	Treatment of exposed tumors that affect the skin, oesophagus, and stomach and are easily accessible for light irradiation.	[55]
	(tetrahydropyranyl methacrylate co-amino ethyl methacrylate)-grafted-mesoporous silica nanoparticles loaded with 5-flurouracil.	Potential applicability in site selective transdermal delivery of chemotherapeutic drugs.	[56]
	MSNs loaded with two ginsenosides: ginsenoside compound K and Rh2.	Potential candidate to load ginsenosides with anti-cancer and anti-inflammatory efficacy.	[57]

**Table 2:** Recent advances on MSNs as brain drug delivery systems.

Application	Structure	Features	References
**Drug Delivery**	MSNs surface coated with Polyamidoamine (PAMAM), polyethylene glycol (PEG) and lactoferrin (Lf).	Lactoferrin-modified NPs, a ligand for brain-targeting drug delivery systems.	[68]
	MSNs surface modified with PEG (PSi NPs) and conjugated with Lf.	Brain drug delivery probe by covalently binding Lf to PSi NPs to achieve receptor-mediated delivery of NPs across the BBB	[69]
**Neurodegenerative Diseases**	MSNs surface coated with a suitable derivative of the arylboronic acids, 3-carboxyphenylboronic acid (MSN-BA). MSN loaded with the dye rhodamine B and capped with human IgG.	MSN based H_2_O_2_ responsive controlled-release system used for Alzheimer’s Disease Treatment	[70]
	Gold nanoparticle-capped mesoporous silica (MSN-AuNPs): a H_2_O_2_-responsive controlled release system for targeted delivery of the metal chelator clioquinol (CQ).	Inhibition of the amyloid-β aggregation and of formation of neurotoxic ROS in the Alzheimer’s disease treatment.	[71]
	MSNs loaded with Rivastigmine hydrogen tartrate, a carbamate-derived reversible cholinesterase inhibitor that is selective for the central nervous system.	NPs used to treat confusion (dementia) related to Alzheimer’s disease and Parkinson’s disease.	[72]
	Polylactic acid (PLA)-coated MSNs (MSNPs), conjugated with a ligand peptide of the low-density lipoprotein receptor (LDLR) and loaded with resveratrol.	A Resveratrol delivery system for the treatment of various central nervous system disorders associated with oxidative stress.	[73]
	MSNs loaded with the phytochemicals curcumin and chrysin.	Nose-to-brain delivery system for the treatment of various central nervous system disorders.	[62]
**Brain Tumor**	Nanosystem modified by RGD (arginine–glycine–aspartate) peptide useful as a carrier of anticancer agents, by using a novel organic selenium compound BSeC as a potential chemotherapeutic agent.	New strategy for the rational design of a tailored nanomedicine with enhanced BBB permeability to treat human brain glioma.	[74]
	MSNs conjugated with cRGD peptide to enhance its cancer targeting effect, and loaded with the antineoplastic drug doxorubicin.	The functionalized nanosystem selectively recognizes glioma cells, inducing apoptosis by triggering ROS overproduction.	[75]
